# Longitudinal Social Network Analysis of Peer, Family, and School Contextual Influences on Adolescent Drinking Frequency

**DOI:** 10.1016/j.jadohealth.2019.03.004

**Published:** 2019-09

**Authors:** Mark McCann, Julie-Ann Jordan, Kathryn Higgins, Laurence Moore

**Affiliations:** a MRC/CSO Social and Public Health Sciences Unit, University of Glasgow, Scotland; b Centre for Evidence and Social Innovation, Queen's University Belfast, Northern Ireland

**Keywords:** Social network analysis, Peer influence, Alcohol use, Parental influence, Stochastic Actor-Oriented Model, Systems model

## Abstract

**Purpose:**

The aim of the study was to identify the mechanisms relating to parental control, adolescent secrecy, and school context that shape patterns of adolescent drinking frequency and appraise the implications for systems-level intervention.

**Methods:**

The Belfast Youth Development Study collected information on friendship networks in schools, alcohol use, and Stattin and Kerr's parental monitoring subscales across 5 years of postprimary school education in annual waves from age 11–15 years. Stochastic Actor-Oriented Models were fitted to 22 schools (N = 3,220) to assess friendship formation and peer influence processes related to drinking frequency and their variation by parental control or child secrecy. Meta-regressions and summary statistic ego-alter selection tables assessed how network and behavior co-evolution varied according to school gender and the proportion of weekly or more frequent drinkers in each school.

**Results:**

Adolescents tended to mimic their peers' drinking levels, and frequent drinkers befriended those who drank similarly to them. Those with high parental control were less likely to befriend low-control peers, whereas low-control pupils were more likely to befriend each other. Adolescents with low-control parents nominated fewer friends in schools with higher proportions of drinking frequently. There was a tendency toward befriending highly secretive peers in boys schools only.

**Conclusions:**

Our results suggest that the optimal strategy for selecting seed nodes in a diffusion of innovations network intervention may vary according to school context, and that targeting family interventions around parent characteristics may modify the wider school network, potentially augmenting network intervention processes.

**Implications and Contribution**

Network analysis explained how peer and family social processes interact to influence adolescent alcohol use patterns and how these vary by school context. These findings suggest implementation strategies for “diffusion of innovation” interventions could differ according to school context, and targeted family intervention components may enhance school network interventions.

Public health interventions that are based on a thorough understanding of the complex social system in which they operate are those most likely to be effective and lead to sustainable change. An appropriate approach to develop systems-level understanding is to take a broad conceptualization of theory, drawing on ideas from different disciplines, to describe the system in question [1]. Using computational models to understand how social interactions between individuals produces system-level patterns [2] can thus help conceptualize the “underlying logic of a potential health promotion intervention” [3] in a complex context, such as the multiple peer and family mechanisms influencing behavior in the adolescent school environment. Modeling may give insights into how the system operates that can plausibly account for contextual variations [4] and shed light on potential mechanisms through which an intervention may have an influence [5], [6]; in particular, how the properties of social networks can be used to diffuse health-improving information [7].

In this article, we use modeling to conceptualize the social system relating to adolescent drinking frequency and discuss the implications for interventions in the school and family context.

While acknowledging that other theories (e.g., social learning theory) could be applied, we have used theories that focus specifically on adolescents and peers and adolescents and family. Erikson's theory of identity development suggests adolescents' self-concept develops according to how others view them at the expense of self-reflection, engendering conformity and mimicry [8]. Bourdieusian theory would frame drinking as a social practice, situated within the drinking behavior, attitudes, and expectations of others [9], [10], which similarly foregrounds the role of others in influencing behavior. Although Bourdieu himself was more concerned with objective class structures rather than intersubjective social structures, the interaction between social structure and individual behavior is still pertinent, particularly for explaining adolescent social structures and health behavior [11].

Previous studies of adolescent drinking using Stochastic Actor-Oriented Models (SAOMs) found evidence for both selection and influence processes; but studies varied in uncovering one, the other, or both processes. There was further variation in whether the processes appeared in early versus late adolescence. The patterns may be due to the use of different alcohol variables: an SAOM for first use of alcohol [12] uncovers a different social process than a model for intensity of use [13], [14], [15], [16], whereas number of previous drinking occasions [17] is dependent on onset age and frequency, thus capturing both processes. Although age of drinking onset is of sociological interest; recent work has demonstrated that it has less relevance for public health than studying the progression toward more risky drinking [18], so we focus on escalating drinking frequency in this article. Koepke suggests that parent influences should decrease through adolescence, and peer influence wane at older ages as individuals become more committed to behavioral aspects of their identity [19]. Prior studies found peer influence emerges in later adolescence [13], so we assessed variation in the social processes with age.

RQ A: Is similar drinking frequency selected for in friendship?
RQ B: Do adolescents modify their alcohol use to mimic their friends?
RQ C: Is secrecy socially transmitted?

Koepke's integrated theory considers the dynamic relations between child identity and parent–child relationship within a family system. From this perspective, parents mediate the relationship between child and society, affording greater social exploration may lead to the social environment having more of an influence on behavioral aspects of self [19]. For example, the observed effect of parental control to reduce drinking [20], [21] may be due to parents preventing the formation of friendships with drinkers, mitigating the social influence of frequent drinking peers. There may also be a family-level mechanism: parents facilitating intergenerational social closure by encouraging their children to form friendships with children whose parents are similar [22]; hence, highly controlled social groups emerge with less drinking opportunities.

RQ D: Does parental control influence friendship formation?
RQ E: Does parental control influence the tendency to befriend drinkers?
RQ F: Does parental control mitigate peer influence on alcohol behavior?

### Secrecy as agency

We operationalize adolescent agency via secrecy to study how individual choices shape the social environment. Adolescent secret-keeping can be seen as two pronged, having positive aspects through creating an independent identity and strengthening social ties to peers, but carrying an increased risk of psychological strain through insufficient parental support, engendering peer *heteronomy* [19] rather than *autonomy* [23], [24]. Hence, we may expect secrecy to be socially desirable and attract friends, or secrecy may engender heteronomy in the form of enhanced drinking mimicry. On the other hand, having many peers who drink often may encourage being secretive, as it facilitates prohibited socializing with drinking peers.

RQ G: Does friends' alcohol use induce secrecy?
RQ H: Are secretive pupils more prone to influence?
RQ I: Is secret keeping selected for in friendship?

### Variation by system context

Contextual features of schools could influence how social processes unfold and thus affect the context dependence of a social intervention [25]. Tilly's theory of durable inequalities defines “opportunity hoarding” as the tendency for groups to become overrepresented in certain roles or resources [26], [27]. Here, we apply it to understand social behavioral roles (e.g., social drinker), which are more salient facets of adolescent social identity than job role. We could expect to see social closure occur or not, dependent on whether alcohol use is ordinary/normative or deviant/subordinate activity in a given context. We propose that identifying asymmetries in the operation of social processes (differential relationships comparing those high vs. low on a given characteristic) can provide novel insight for potential intervention, and we scrutinize ego-alter selection tables with such asymmetry in mind. Alcohol is associated with Western masculine norms [28]. Prodrinking injunctive norms (i.e., a broader perceived societal norm) in a boys-only context would make drinking more desirable and more mimicked than in a mixed gender school context. Schools with a greater proportion of drinkers set a prodrinking descriptive norm (i.e., a norm in the immediate environment), eliciting greater mimicry than in less frequent drinking settings. SAOM in a sample of U.S. schools found little evidence of contextual variation [29], but UK schools research has found that the school culture and environment could influence social processes relating to peer groups and behavioral norms at the school and peer group level [30].

RQ J: Does school gender influence selection and influence processes (SIP)?
RQ K: Does the proportion of frequent drinkers in the school influence selection and influence processes?

### Aims and research questions

The aim of the article was to identify theory-informed mechanisms acting across multiple levels of the social system (adolescent, school peer, and family) that influence adolescent drinking frequency. In support of this aim, we will answer research questions A to K above and discuss the findings in relation to the design of health interventions.

## Methods

### Data collection

The Belfast Youth Development Study (BYDS) is a school-based study of drug and alcohol use. All the post primary schools in Belfast and two intermediate townlands in Northern Ireland were invited to take part, 71% agreed. All students in participating schools were surveyed in their first year of post primary education in the academic year 2000/2001 and annually thereafter for 5 years of compulsory schooling. The study is described in detail elsewhere [31]. The participant count and response proportions were wave 1: 3,834 (87%); wave 2: 4,343 (83%); wave 3: 4,522 (86%); wave 4: 3,965 (76%); wave 5: 3,830 (74%). Nonresponse was around 5% refusals, and the remainder due to absence on survey day and follow-up visit.

### Social networks

In each year of the survey, participants were asked to name their best friend and up to nine other friends in their school year. Social network information was collected from 42 schools. Our analysis used schools with <20% missing data on friendship nominations.

We included each pupil's gender and free school meal eligibility as main effect predictors of behavior and dyadic covariates to model the tendency for friendship ties to form between similar peers.

### Respondent characteristics

The alcohol frequency measure was based on questions relating to ever used alcohol (yes/no) and frequency of drinking asked in each year of the study. The four-category variable was never, infrequently (has drunk alcohol, but does not regularly), monthly, and weekly or more frequently. The parental control subscale and child secrecy items within the child disclosure subscale of the Stattin and Kerr parental monitoring scales were used in analysis (adolescent reported. Described elsewhere [21] and in Appendix). Higher scores indicate more parental knowledge, that is, higher control and lower secrecy.

### Model parameters

A set of core processes determine much of the structure of social networks across contexts, and these were included in all models (Appendix). Model parameters relating to hypotheses can be characterized as ego effects (relating to a characteristic of the individual that may influence that individual's behavior), alter effects (characteristics of others that may influence an individual's behavior), and similarity effects (the similarity between ego and alter or a set of alters). We assessed SIP on alcohol use by including an alter effect, ego effect, and a similarity effect for tie formation; and for influence, a total similarity effect for drinking frequency. Total similarity models a tendency to drink more frequently depending on the absolute number of frequent drinkers among one's social connections. The average similarity effect can depict theories around descriptive norms [32], [33], [34], [35], knowing a greater proportion of drinkers elicits a greater perceived behavioral norm around drinking, assuming a stable effect of peer group size. Total similarity additionally accounts for greater instrumental opportunities to obtain alcohol from a larger peer group (NB. alternative peer influence mechanisms may plausibly explain a positive total similarity parameter estimate).

We included parameters to determine if parental behavior influences friendship networks directly by including alter, ego, and similarity effects for control. We included parameters to assess if parental control influences the social processes within schools while accounting for the reverse process where drinking changes parental control [21]. The interaction between parental control and “similar drinking to peers” on tie formation and the interaction between parental control and “similar drinking to peers” on drinking behavior.

We modeled whether the social environment drives secretive behavior directly via mimicking levels of peer secrecy or indirectly through peer's alcohol use incentivizing secret-keeping using an alcohol average similarity parameter to predict secrecy. Finally, to assess whether secretive pupils respond differently to their peers, we included the interaction between secrecy and total alcohol similarity.

### School context

Each school's gender status (coeducational, girls only, or boys only) and overall drinking prevalence (percentage of Year 3 pupils reporting alcohol use weekly or more frequently) were entered as predictors of variation in parameters in a meta-regression. School results were combined with empirical Bayes random effects meta-analysis using the R Package metafor. Where analyses suggested there were variations in selection processes, we produced summary ego-alter selection tables by school type. This allowed us to scrutinize the between-school variation in asymmetries in friendship formation and “opportunity hoarding” patterns that would be difficult to see by looking across separate model results.

### Model building

All structural, covariate, and hypothetical parameters were entered into a full model, and these models were updated using previous results to improve convergence. In one school, we fixed one parameter at zero to enable convergence; otherwise, we retained nonsignificant parameters to conduct meta-analyses and time trend tests. We assessed time heterogeneity in our research question parameters. There was some evidence that the influence of alcohol, parental control, and secrecy on friendship ties may vary across the study years, but not for influence processes. This contrasts with those of previous studies [15], and Erikson's theory [8], see Appendix for full results.

We fitted unconditional methods of moments estimation for SAOMs using the R Package RSiena [36]. Total convergence below .25, parameter convergence below .1, and violin plots for indegree, outdegree, and triad censuses suggested that model fit was adequate for all networks. SAOMs operate by simulating pupil behaviors (e.g., “reciprocate a friendship tie,” “increase drinking toward their friends’ average drinking level”) at microsteps between observation periods. These “behavioral rules” are varied across many simulations, the structure of simulated data compared with observed data, until a set of behavioral rules that closely recreate the observed structure are obtained. Model output provides coefficients representing probability of individual decisions regarding friendship or behavior; and all parameters are conditional on all others within the model (e.g., I befriend those drinking similarly, over and above befriending those of the same gender). Further information is available at stats.ox.ac.uk/∼snijders/siena.

## Results

There were nine coeducational schools, eight girls schools, and five boys schools in the final analysis with a total of 3,220 pupils in the sample. Table 1 describes the included schools by school type, school size varied from 60 to 210 pupils. Around 50% of coeducational school pupils were female, and the proportion of pupils taking free school meals varied widely and declined over time. Weekly drinking increased over time, from below 5% to above 60%. Some schools dropped out in later years; hence, the sample means do not reflect the overall time trend.

**Table 1:** School summary statistics by study wave and school type

Network characteristics	Year 1	Year 2	Year 3	Year 4	Year 5
Coeducational schools					
Schools in year	8	7	9	7	6
Pupils in year	133.4 (73,203)	135.6 (72,208)	132.3 (73,207)	133.4 (73,210)	130.4 (75,210)
% Present	.87 (.78, .93)	.85 (.79, .91)	.89 (.82, .94)	.88 (.72, .98)	.79 (.54, .92)
% Female	.48 (.39, .58)	.49 (.42, .56)	.48 (.40, .55)	.49 (.43, .54)	.49 (.40, .56)
% Free meals	.18 (.00, .67)	.16 (.01, .60)	.19 (.01, .57)	.17 (.02, .57)	.04 (.00, .11)
% Drinking weekly	.05 (.00, .10)	.11 (.03, .17)	.20 (.11, .34)	.31 (.11, .58)	.38 (.21, .49)
Parental control % high control tertile	.62 (.40, .81)	.53 (.38, .70)	.49 (.33, .67)	.45 (.26, .61)	.47 (.38, .55)
Child secrecy % low secrecy tertile	.67 (.53, .78)	.55 (.35, .70)	.50 (.34, .67)	.52 (.36, .67)	.38 (.28, .47)
Girls only schools					
Schools in year	6	7	8	7	7
Pupils in year	114.7 (93,150)	110.8 (75,152)	110.0 (75,150)	110.0 (75,148)	109.0 (73,150)
% Present	.90 (.86, .93)	.88 (.79, .94)	.90 (.85, .93)	.90 (.84, .97)	.84 (.80, .92)
% Free meals	.09 (.01, .32)	.18 (.02, .47)	.16 (.02, .43)	.17 (.02, .46)	.16 (.01, .38)
% Drinking weekly	.03 (.01, .08)	.11 (.02, .16)	.24 (.13, .43)	.37 (.27, .54)	.47 (.38, .60)
Parental control % high control tertile	.68 (.54, .74)	.54 (.36, .73)	.53 (.35, .68)	.49 (.29, .65)	.46 (.32, .58)
Child secrecy % low secrecy tertile	.71 (.60, .79)	.55 (.45, .69)	.52 (.30, .74)	.47 (.24, .61)	.39 (.25, .49)
Boys only schools					
Schools in year	5	5	4	4	2
Pupils in year	157.8 (109,200)	156.6 (107,198)	156.0 (102,198)	155.8 (102,200)	153.4 (101,195)
% present	.89 (.84, .92)	.90 (.88, .93)	.89 (.85, .94)	.93 (.87, 1.00)	.81 (.69, .91)
% Female	—	—	—	—	—
% Free meals	.30 (.06, .64)	.29 (.06, .65)	.17 (.05, .37)	.16 (.07, .35)	.08 (.06, .09)
% Drinking weekly	.06 (.04, .11)	.14 (.10, .16)	.24 (.21, .29)	.32 (.18, .38)	.36 (.35, .36)
Parental control % high control tertile	.47 (.29, .66)	.40 (.35, .47)	.45 (.41, .52)	.36 (.23, .45)	.41 (.39, .43)
Child secrecy % low secrecy tertile	.57 (.49, .70)	.52 (.46, .58)	.46 (.39, .52)	.38 (.36, .41)	.30 (.30, .30)
Total					
Schools in year	19	19	21	18	15
Pupils in year	133.90 (73,203)	131.32 (72,208)	129.41 (73,207)	130.00 (73,210)	127.86 (73,210)
% Present	.88 (.78, .93)	.87 (.79, .94)	.89 (.82, .94)	.90 (.72, 1.00)	.81 (.54, .92)
% Female	.52 (.00, 1.00)	.55 (.00, 1.00)	.59 (.00, 1.00)	.58 (.00, 1.00)	.66 (.00, 1.00)
% Free meals	.18 (.00, 67)	.20 (.01, 65)	.18 (.01, .57)	.17 (.02, .57)	.10 (.00, .38)
% Drinking weekly	.05 (.00, .11)	.12 (.02, .17)	.22 (.11, .43)	.34 (.11, .58)	.42 (.21, .60)
Parental control % high control tertile	.60 (.29, .81)	.50 (.35, .73)	.50 (.33, .68)	.45 (.23, .65)	.46 (.32, .58)
Child secrecy % low secrecy tertile	.66 (.49, .79)	.54 (.35, .70)	.50 (.30, .74)	.47 (.24, .67)	.37 (.25, .49)

School totals are lower than the 22 school whole sample because school participation varied from year to year.

The network structural parameters were broadly comparable across all schools, with expected tendencies for gender assortativity, reciprocal friendship, popularity effects, and evidence of a similarity preference according to free school meal eligibility (Appendix).

Table 2 shows the results of meta-analyses for the parameters relating to RQs A to H, combining results from all 22 schools (Table A2, Appendix for a findings summary). There was strong evidence for friendship selection based on alcohol similarity (A) and for peer influence on alcohol behavior (B). There was evidence of selection based on parental control (D) and weak evidence based on child secrecy (C). Parental control does not otherwise appear to influence friendship nominations; however, secretive pupils received more friend nominations. Regarding ego's drinking behavior, there was no evidence of an interaction between peer influence (total alcohol similarity) and parental control (F) or peer influence and secrecy (H). Regarding friendship nomination, there was no evidence for interactions between alcohol peer selection (average alcohol similarity) and control (E). Controlling for the direct effect of individual secrecy, peers' drinking did not appear to influence ego's secrecy (G).

**Table 2:** Meta-analysis of research question parameters and meta-regression by school gender and drinking prevalence

Parameters	Interpretation of a positive estimate	Meta-analysis	Meta-regression			
			Intercept	Boys	Girls	% Frequent Drinkers
		Log odds (95% CI)	Log odds (95% CI)	Log odds (95% CI)	Log odds (95% CI)	Log odds (95% CI)
alc.beh alter	I befriend those who drink more	**.03 (.01, .05) *p* = .004**	.05 (−.03, .13) *p* = .25	−.05 (−.11, .01) *p* = .12	.00 (−.06, .05) *p* = .93	−.02 (−.42, .38) *p* = .93
alc.beh ego	If I drink more, I befriend more often	0.01 (−.03, .05) *p* = .56	.10 (−.03, .24) *p* = .14	−.01 (−.12, .09) *p* = .79	.06 (−.04, .16) *p* = .23	−.5 (−1.17, .17) *p* = .14
alc.beh similarity	I befriend those drinking similarly	**.35 (.27, .43) *p* < .001**	**.60 (.31, .89) p ≤ 001**	.04 (−.19, .27) *p* = .73	−.08 (−.28, .11) *p* = .40	−1.11 (−2.55, .33) *p* = .13
sec.beh alter	I befriend those who are more disclosive	**−.05 (−.08, −.02) *p* < .001**	−.10 (−.20, .01) *p* = .07	**−.08 (−.16, .00) *p* = .05**	**−.07 (−.14, .01) *p* = .07**	.38 (−.12, .88) *p* = .14
sec.beh ego	If I am more disclosive, I befriend more often	.00 (−.06, .05) *p* = .95	.03 (−.17, .24) *p* = .75	−.03 (−.2, .14) *p* = .72	.02 (−.12, .16) *p* = .81	−.17 (−1.14, .81) *p* = .74
sec.beh similarity	I befriend those who are similarly secretive	**.09 (−.01, .19) *p* = .08**	.13 (−.24, .5) *p* = .50	.09 (−.22, .39) *p* = .58	.00 (−.25, .25) *p* = 1.00	−.28 (−2.02, 1.46) *p* = .75
con.beh alter	I befriend those with stricter parents	−.01 (−.03, .02) *p* = .69	−.06 (−.17, .04) *p* = .24	.06 (−.02, .14) *p* = .14	.00 (−.07, .07) *p* = .93	.21 (−.32, .74) *p* = .44
con.beh ego	If my parents are more strict, I befriend more often	−.03 (−.08, .03) *p* = .31	**.15 (−.03, .33) *p* = .1**	.00 (−.14, .14) *p* = .99	−.01 (−.14, .13) *p* = .93	**−.80 (−1.67, .06) *p* = .07**
con.beh similarity	I befriend those with similar parents	**.12 (.03, .20) *p* = .008**	.12 (−.18, .42) *p* = .43	−.17 (−.42, .07) *p* = .17	−.01 (−.23, .21) *p* = .92	.20 (−1.29, 1.70) *p* = .79
Con.beh * Alc sim	My high control parents prevent me befriending similar peers	.09 (−.10, .27) *p* = .36	.20 (−.50, .90) *p* = .57	−.3 (−.87, .27) *p* = .30	−.21 (−.72, .30) *p* = .42	.04 (−3.50, 3.58) *p* = .98
alc.beh: total similarity	I mimic my peers' drinking	**.46 (.38, .54) *p* < .001**	**.63 (.34, .92) *p* ≤ .001**	−.01 (−.25, .24) *p* = .96	−.01 (−.21, .19) *p* = .9	−.83 (−2.38, .72) *p* = .30
Alc: con * Alc totsim	My strict parents inhibit my drinking mimicry	.10 (−.09, .30) *p* = .30	−.28 (−.98, .43) *p* = .44	.20 (−.37, .77) *p* = .48	**.41 (−.08, .91) *p* = .10**	.94 (−2.86, 4.73) *p* = .63
Alc: Sec * Alc totsim	My low secrecy inhibits my drinking mimicry	.08 (−.07, .24) *p* = .29	−.34 (−.88, .19) *p* = .21	.11 (−.34, .56) *p* = .63	.06 (−.31, .43) *p* = .75	1.81 (−.87, 4.48) *p* = .19
sec.beh average similarity	I mimic my peers' secrecy	**1.20 (.65, 1.75) *p* < .001**	**1.7 (−.22, 3.62) *p* = .08**	−2.06 (−3.92, −.2) *p* = .03	−.41 (−1.63, .82) *p* = .52	−.4 (−9.8, 9.01) *p* = .93
sec.beh: Alter's average drinking^a^	My peers' drinking makes me more secretive	−.06 (−.25, .12) *p* = .48	**−.67 (−1.17, −.16) *p* = .01**	−.44 (−.98, .1) *p* = .11	.15 (−.18, .49) *p* = .36	2.81 (.38, 5.23) *p* = .02
Con: effect from Alc	My drinking raises my parent's control	**−.25 (−.33,−.18) *p* < .001**	**−.54 (−.81, −.27) *p* ≤ .001**	−.01 (−.21, .18) *p* = .89	.00 (−.18, .17) *p* = .97	**1.28 (.01, 2.54) *p* = .05**

Bold type denotes *p* value below .1.

Meta-regression intercept: the estimated parameter value for a coeducational school with the study sample mean proportion of frequent drinkers.

Meta-analysis parameter: the pooled summary parameter across all schools in the sample.

CI = confidence interval.

a Parameter excluded from model for girls school 4”.

### Differences by school context

There was some evidence that selection by secrecy was stronger in single gender compared with coeducational schools (J); high parental control reduced outgoing friendship ties in schools with higher proportions of frequent drinkers (K); and, that adolescent drinking was more strongly associated with a reduction in parental control in schools with a higher proportion of drinkers (K).

The coefficients in the ego-alter selection tables (Table 3, Table 4, Table 5) represent odds ratios (OR; 95% confidence intervals [CIs]) of forming a friendship tie according to friendship sender and receiver characteristics, compared with two individuals at the mean level of the characteristic. Looking first at alcohol (Table 3), there is a strong tendency not to befriend individuals with different drinking patterns; a nondrinker is around 17% less likely to befriend a frequent drinker, and a frequent drinker 21% less likely to befriend a nondrinker. Looking at drinking similarity, nondrinkers are no more likely to befriend each other, whereas a frequent drinker is 19% more likely (95% CI 10%–30%) to befriend a similarly frequent drinker.

**Table 3:** All schools: odds ratios (confidence intervals) of friendship tie by ego and alter drinking

		Alter—Receiver's drinking			
		None	Rarely	Monthly	Frequently
Ego Sender's drinking	None	1.04 (.98, 1.11)	.96 (.91, 1.02)	**.89 (.82, .97)**	**.83 (.75, .92)**
	Rarely	**.95 (.91, .98)**	**1.09 (1.06, 1.12)**	1.02 (.98, 1.05)	.94 (.89, 1.00)
	Monthly	**.86 (.82, .91)**	1.00 (.96, 1.03)	**1.14 (1.09, 1.18)**	**1.06 (1.01, 1.11)**
	Frequently	**.79 (.73, .87)**	**.90 (.84, .97)**	1.04 (.97, 1.12)	**1.19 (1.10, 1.30)**

Odds of a tie relative to two friends with school mean level of alcohol frequency.

Bold type denotes non-overlapping CI for the linear combination of ego, alter, and similarity parameters.

**Table 4:** All schools: parental control ego alter selection table

		Alter—Receiver's control		
		Low control	Moderate	High control
Ego Sender's control	Low control	**1.11 (1.01, 1.21)**	1.03 (.97, 1.10)	.97 (.89, 1.05)
	Moderate	.98 (.95, 1.02)	1.06 (1.01, 1.11)	.99 (.95, 1.02)
	High control	**.90 (.83, .97)**	.95 (.91, 1.00)	1.02 (.99, 1.05)

Odds of a tie relative to two friends with school mean level of control.

Bold type denotes non-overlapping CI for the linear combination of ego, alter, and similarity parameters.

**Table 5:** Stratified by school type: secrecy ego alter selection table

		Alter—Receiver's secrecy		
		Secretive	Moderate	Disclosive
All schools				
Ego Sender's secrecy	Secretive	1.07 (.95, 1.20)	1.01 (.94, 1.08)	.94 (.87, 1.01)
	Moderate	1.04 (1.00, 1.09)	1.04 (.99, 1.10)	.96 (.94, 1.00)
	Disclosive	1.02 (.94, 1.10)	1.01 (.97, 1.05)	1.00 (.96, 1.04)
Coeducational schools				
Ego Sender's secrecy	Secretive	.99 (.84, 1.15)	.99 (.90, 1.09)	.96 (.87, 1.05)
	Moderate	1.01 (.95, 1.06)	1.02 (.95, 1.09)	1.00 (.97, 1.03)
	Disclosive	1.01 (.91, 1.12)	1.02 (.96, 1.09)	1.02 (.96, 1.08)
Girls schools				
Ego Sender's secrecy	Secretive	1.10 (.88, 1.37)	.99 (.87, 1.13)	.91 (.76, 1.09)
	Moderate	1.06 (.98, 1.15)	1.04 (.93, 1.17)	.94 (.88, 1.01)
	Disclosive	1.02 (.85, 1.23)	1.01 (.92, 1.10)	.99 (.93, 1.06)
Boys schools				
Ego Sender's secrecy	Secretive	**1.24 (1.11, 1.39)**	1.06 (.93, 1.21)	.91 (.77, 1.08)
	Moderate	1.10 (.99, 1.22)	**1.10 (1.07, 1.14)**	.93 (.91, .97)
	Disclosive	.98 (.89, 1.08)	.98 (.95, 1.01)	.98 (.83, 1.14)

Odds of a tie relative to two friends with school mean level of secrecy.

Bold type denotes non-overlapping CI for the linear combination of ego, alter, and similarity parameters.

Parental control's influence on friendship formation shows a different pattern (Table 4). Pupils experiencing high control are less likely to befriend low (OR .90; 95% CI .83, .97) or moderate (.95; 95% CI .91, 1.00) control peers, whereas low-control peers are more likely to befriend those similar to themselves (OR 1.11; 95% CI 1.01, 1.21). This asymmetry may reflect an opportunity hoarding process: low-control peers coalesce into friendship groups, potentially sharing information and behavior patterns that will not be transmitted to high-control peers.

Considering all schools combined, tie formation does not appear strongly patterned by adolescent secrecy, but the meta-regression identified school variation in tie formation by secrecy. Stratified by school gender (Table 5), secrecy plays little role in friendship formation in coeducational schools, there is a nonsignificant trend toward homophily among secretive pupils in girls-only schools, and a strong tendency toward homophily among moderate and high-secrecy pupils in boys-only schools.

## Discussion

This article used complex systems methods to scrutinize how school, peer, and family environments relate to adolescent drinking, making two novel contributions. First, we have uncovered elements of family and school context influencing the formation of friendship groups and drinking patterns; second, we have demonstrated how SAOMs, meta-regression, and meta-analysis of ego-alter selection tables help understand opportunity hoarding and the formation of inequalities between groups in social network data, which has implications for how to implement network interventions.

Individual agency (peer selection) and social structure (peer influence) both play a role to explain the clustering of drinking behavior within schools, although friend selection is driven by prodrinking rather than nondrinking peers. Intergenerational closure–befriending peers with similar parents [22] do not appear directly in relation to control, but low-control parents may dissuade their children befriending low parental control peers. There is no suggestion that the adolescent or family characteristics magnify or mitigate alcohol selection and influence processes, but features of schools may influence both peer network and family dynamics. Adolescent boys exert greater agency to create secretive peer groups in single compared with mixed gender schools, and frequent drinking adolescents more rapidly experience reduced parental control when in schools with higher drinking rates.

If we aim to intervene, then alcohol use is better studied as a socially situated practice rather than a health risk outcome [10]. Person-level predictors of drinking fail to capture features of the broader environment that determine the behavior of groups. Although our findings confirm that adolescents simultaneously act to change and are shaped by their social environment [22], [29], we have uncovered two novel insights for intervention design.

First, the school context may change the reach of peer-led school interventions, although the change processes may be the same. Drinking mimicry did not vary according to our theoretical expectations around normative differences related to school gender or school-level drinking, suggesting diffusion of innovation interventions [7] could have a similar change mechanism across contexts. On the other hand, the structure of friendship groups differs by school gender. This means that the pathways through which intervention information diffuses from a “seed” node (a network member that receives intervention to diffuse content to others) to a “need” node (network member that does not receive intervention but would benefit from its content) will vary. If boys schools more often have dense and high risk (secretive) friendship clusters, this suggests that network diffusion interventions [7], [37] will encounter “harder to reach” and “harder to treat” groups. Intervention success depends on identifying seed nodes within these groups and acknowledging that the outside-school risk profile for these cliques differs from the wider network.

Secondly, addressing low-control parenting practices may interrupt the formation of high-risk school cliques, whereas raising moderate or high levels of parental control will have little spillover effect on other peers. This suggests that a “universal school” (focus on all pupils) but “targeted/proportionate community” (focus on families with higher risk profile) intervention could be more effective and resource efficient [38].

Our meta-analysis of ego-alter selection tables allows us a better understanding of the processes around social closure than is possible by looking at summary statistics for tie formation parameters. Theories relating to community and social control would suggest that a friend's parents could influence one's own behavior in adolescence [22], but our findings suggest adolescent secrecy plays a greater role in shaping the peer environment than parental behavior. Low-control parents do not attempt or are unsuccessful in inducing intergenerational “closure” (22) by connecting to parents with similar practices via their children's peer group, whereas low-control adolescents—those for whom parents are not influencing their social routines—seem to actively coalesce into friendship groups. Notably, this pattern appears over and above social clustering according to alcohol use and deprivation.

### Strengths and limitations

The five waves of network data in the Belfast Youth Development Study gave the opportunity to study social influence from the move into postprimary school throughout the transition into adolescence, the time when social ties to peers becomes most salient for determining behavior. The large number of schools allowed us to study both generalizable processes as well as variation with school and family context. SAOMs also provide advantages in dealing with nonindependent observations, and the ability to study selection, influence, and contextual processes. Our study also has limitations. Our parental monitoring measures were adolescent rather than parent reported and thus could be biased according to attitudinal, social, or demographic variation or other individual differences. Second, we are unable to explore our postulated theoretical processes in depth using quantitative data alone. SAOM provides a method to move beyond Bhaskar's *empirical*—simply assessing patterns of change in the survey data—and instead to use simulation of *real* processes (or potential versions of the processes) that generate observed change in our data, but we have insufficient information to explore *actual* mechanisms of change and explanation for change by school context [39]. Further network research integrating a qualitative longitudinal component would open up new possibilities to integrate explanatory theories and models: personal accounts of how social groups form, shared social activities, and the relationship between peers and parents would give a more meaningful interpretation of how SAOM microsteps relate to decisions around interpersonal relationships and school-level patterns. Finally, changes in society in relation to phone and social media use since data collection could plausibly change the nature of social influence processes. Research on new cohorts could assess such changes.

Although there is no evidence that parenting practice changes peer processes directly [22], efforts to reduce risky drinking patterns in adolescence could usefully focus on family plus peer interventions. Our findings suggest that, in relation to the family component, effective targeting of families with the lowest levels of parental control is necessary to induce a change in the school peer social environment. For the peer component, the most appropriate pupils to recruit for peer-led interventions may be context dependent; boys-only schools may be more amenable to a segmentation approach, whereas girls and mixed gender schools require less targeting of clusters and could identify key players [7]. Finally, high levels of secret-keeping drive the formation of social groupings, but secretive pupils are not in themselves more prone to alcohol influence. Interventions to build efficacy and skills in productive relationship building and information sharing, with parents and with peers, could act as a conduit to elicit “network induction” effects, altering the formation of positive social connection without necessarily altering social transmission of risk behavior. More generally, the findings of this study highlight the fact that adolescent health interventions should pay greater attention to the agency young people enact to shape their family and peer environment.
