# Supplementary material for: Longitudinal Social Network Analysis of Peer, Family, and School Contextual Influences on Adolescent Drinking Frequency
Source: J Adolesc Health. 2019 Sep;65(3):350–8. doi: 10.1016/j.jadohealth.2019.03.004 (PMC6710020; doi:10.1016/j.jadohealth.2019.03.004)
Supplement: Appendix [file mmc1.docx]

**Appendix**

Items from the Stattin & Kerr parental monitoring subscales were used for analysis. The parental control questions contained items including: ‘do you need to have your parents’ permission to stay out late on a weekday evening’, and ‘if you have been out late one night do your parents require you to explain what you did and who you were with?’

The child disclosure subscale has two dimensions, one relating to active disclosure, sharing information and having discussions with their parents, and another dimension relating to secrecy actively withholding information which may occur independently of whether or not they discuss non-secret issues. Based on the author’s previous study which found little association of active disclosure with alcohol use, we focussed on the active secrecy items of the subscale.

The secrecy subscale included the questions ‘do you keep a lot of secrets from your parents about what you do in your free time?’ and ‘do you hide a lot from our parents about what you do during nights and weekends?’.

These items were answered on a five point likert scale in years 2 to 5: ‘Almost never or never true’, ‘not very often true’, ‘sometimes true’, ‘often true’, and ‘almost always or always true’. In year one, they were answered in yes/no scale.

Table A1 contains reliability measures for these items over the five waves.

Table A1: Reliability measures for parental monitoring sub scales

| **Scale** | **Cronbach’s alpha** |
| --- | --- |
|  |  |
| **Adolescent secrecy** |  |
| **Year 1** | **0.69** |
| **Year 2** | **0.84** |
| **Year 3** | **0.86** |
| **Year 4** | **0.89** |
| **Year 5** | **0.61** |
|  |  |
| **Parental Control** |  |
| **Year 1** | **0.69** |
| **Year 2** | **0.84** |
| **Year 3** | **0.84** |
| **Year 4** | **0.85** |
| **Year 5** | **0.85** |
|  |  |

Table A2: Key for model coefficients, questions, and deductions from the model results

| Parameter | Research question | Deduction |
| --- | --- | --- |
| alc.beh alter | A. Is similar drinking frequency selected for in friendship? | Dissimilarity is avoided, frequent drinkers befriend more often than non-drinkers |
| alc.beh ego |  |  |
| alc.beh similarity |  |  |
| sec.beh alter | RQ I. is secret keeping selected for in friendship? | Highly secretive pupils befriend each other in boys schools, no influence on friendships in coeducational schools |
| sec.beh ego |  |  |
| sec.beh similarity |  |  |
| con.beh alter | D. Does parental control influence friendship formation? | Low control pupils befriend each other, while high control pupils tend not to befriend low control peers |
| con.beh ego |  |  |
| con.beh similarity |  |  |
| con.beh ego x alc.beh similarity | E. Does parental control influence the tendency to befriend drinkers? | No |
| alc.beh total similarity | B. Do adolescents modify their alcohol use to mimic their friends? | Yes |
| alc.beh: effect from con.beh  x alc.beh total similarity | F. Does parental control mitigate peer influence? | No |
| alc.beh: effect from sec.beh  x alc.beh total similarity | H. Are secretive pupils more prone to influence? | No |
| sec.beh average similarity | C. Is secrecy socially transmitted? | Yes |
| sec.beh: Alter’s average drinking | G. Does friends’ alcohol use induce secrecy? | Not directly (indirectly via alter’s secrecy) |
| con.beh: effect from alc.beh | To control for reverse causation of control’s influence on alcohol use: Does adolescent drinking influence parental control? | Frequent drinking reduces parental control, particularly in more frequent drinking schools |

Table A3:Coeducational Schools: log odds for network dynamics (forming a friendship tie) or behaviour dynamics (step increase in behaviour level)

| Effect | Coed 1 | Coed 2 | Coed 3 | Coed 4 | Coed 5 | Coed 6 | Coed 7 | Coed 8 | Coed 9 |
| --- | --- | --- | --- | --- | --- | --- | --- | --- | --- |
| Network Dynamics |  |  |  |  |  |  |  |  |  |
| constant sch4nets rate (period 1) | 85.039 | 17.7 | 12.34 | 46.539 | 23.987 | 18.688 | 32.38 | 17.996 | 15.392 |
| constant sch4nets rate (period 2) | 37.569 | 24.403 | NA | 20.942 | 15.868 | 16.484 | 20.706 | 16.085 | 25.858 |
| constant sch4nets rate (period 3) | 28.942 | 36.448 | NA | 37.52 | NA | 21.506 | 12.24 | 43.013 | 24.147 |
| constant sch4nets rate (period 4) | 20.681 | 22.241 | NA | NA | NA | 16.518 | NA | 18.517 | NA |
| outdegree (density) | -2.059*** | -2.056*** | -1.636*** | -1.917*** | -2.217*** | -2.508*** | -1.764*** | -2.686*** | -1.939*** |
| Reciprocity | 2.356*** | 2.429*** | 2.667*** | 2.161*** | 2.386*** | 2.115*** | 2.174*** | 2.325*** | 2.201*** |
| transitive triplets | 0.464*** | 0.470*** | 0.731*** | 0.465*** | 0.530*** | 0.386*** | 0.445*** | 0.444*** | 0.471*** |
| transitive recipr. Triplets | -0.332*** | -0.362*** | -0.522*** | -0.329*** | -0.401*** | -0.234*** | -0.310*** | -0.323*** | -0.375*** |
| indegree – popularity | 0.007 | 0.027*** | 0.055 | 0.002 | 0.021 | 0.024** | 0.003 | 0.014** | 0.001 |
| outdegree – popularity | -0.105*** | -0.118*** | -0.203* | -0.112*** | -0.108*** | -0.072*** | -0.132*** | -0.081*** | -0.103** |
| outdegree – activity | 0.026* | 0.024* | 0.004 | 0.037** | 0.011 | 0.054*** | 0.029* | 0.068*** | 0.025† |
| gender.dyadcovar | 0.495*** | 0.188*** | 0.375* | 0.174** | 0.485*** | 0.150*** | 0.361*** | 0.283*** | 0.255** |
| meal.dyadcovar | 0.029 | 0.056 | 0.249 | 0.410** | 0.054 | -0.269† | 0.068 | 0.039 | 0.208** |
| alc.beh alter | 0.015 | 0.057† | 0.206 | 0.100† | 0.058 | 0.068† | 0.034 | 0.061† | 0.029 |
| alc.beh ego | 0.04 | 0.021 | 0.118 | 0.014 | 0.067 | 0.046 | 0.058 | 0.028 | 0.009 |
| alc.beh similarity | 0.352** | 0.421** | 1.458 | 0.503* | 0.19 | 0.409** | 0.477 | 0.426** | 0.36 |
| sec.beh alter | 0.044 | 0.017 | 0.137 | 0.008 | 0.007 | 0.018 | 0.042 | 0.07 | 0.043 |
| sec.beh ego | 0.044 | 0.087 | 0.008 | 0.046 | 0.177† | 0.136 | 0.031 | 0.095 | 0.19 |
| sec.beh similarity | 0.049 | 0.161 | 0.311 | 0.154 | 0.062 | 0.072 | 0.05 | 0.225† | 0.521 |
| con.beh alter | 0.048 | 0.01 | 0.119 | 0.054 | 0.08 | 0.061 | 0.029 | 0.017 | 0.045 |
| con.beh ego | 0.110† | 0.071 | 0.096 | 0.023 | -0.281* | 0.085 | 0.113 | 0.192** | 0.016 |
| con.beh similarity | 0.008 | 0.117 | 0.643 | 0.147 | 0.211 | 0.347* | 0.096 | 0.103 | 0.44 |
| int. con.beh ego x alc.beh similarity | 0.015 | 0.319 | 2.479* | 0.391 | 1.175 | 0.43 | 0.749 | 0.383 | 0.061 |
| Behaviour Dynamics |  |  |  |  |  |  |  |  |  |
| rate alc.beh (period 1) | 1.56 | 1.993 | 1.653 | 1.196 | 2.425 | 1.628 | 2.925 | 1.061 | 1.84 |
| rate alc.beh (period 2) | 1.37 | 1.507 | NA | 1.405 | 2.514 | 1.527 | 1.677 | 1.072 | 2.435 |
| rate alc.beh (period 3) | 1.042 | 1.156 | NA | 1.871 | NA | 1.114 | 2.482 | 1.197 | 1.298 |
| rate alc.beh (period 4) | 1.029 | 1.062 | NA | NA | NA | 1.136 | NA | 1.243 | NA |
| alc.beh linear shape | 0.760* | 0.568* | 0.899 | 0.584*** | 0.896*** | 0.533** | 0.743** | 0.309* | 0.348 |
| alc.beh quadratic shape | 0.276* | 0.213† | 0.077 | 0.183 | 0.412* | 0.201† | 0.217 | 0.308** | 0.255 |
| alc.beh total similarity | 0.557* | 0.559*** | 0.233 | 0.384† | 0.494† | 0.463** | 0.586* | 0.494*** | 0.345 |
| alc.beh: effect from gender.covar | 0.072 | 0.043 | 0.371 | 0.178 | 0.317 | 0.16 | 0.301 | 0.061 | 0.46 |
| alc.beh: effect from sec.beh | 0.3 | 0.674 | 0.213 | 0.347 | 0.133 | 0.062 | 0.765 | 0.663 | 0.606 |
| alc.beh: effect from con.beh | 0.518 | 0.514 | 0.369 | 0.347 | 0.115 | 0.503 | 0.341 | 0.368 | 0.438 |
| alc.beh: effect from meal.covar | 0.616 | 0.38 | 0.515 | 0.978 | 0.263 | 0.404 | 0.202 | 0.418 | 0.191 |
| alc.beh: effect from con.beh x alc.beh total similarity | 0.399 | 0.044 | 0.613 | 0.068 | 0.086 | 0.323 | 0.085 | 0.159 | 0.34 |
| alc.beh: effect from sec.beh x alc.beh total similarity | 0.398 | 0.172 | 0.142 | 0.128 | 0.098 | 0.038 | 0.102 | 0.236 | 0.61 |
| rate sec.beh (period 1) | 1.724 | 2.083 | 2.123 | 2.042 | 1.925 | 1.702 | 1.656 | 1.742 | 2.839 |
| rate sec.beh (period 2) | 1.442 | 2.012 | NA | 2.054 | 1.879 | 1.859 | 1.653 | 1.724 | 2.858 |
| rate sec.beh (period 3) | 1.451 | 1.795 | NA | 1.399 | NA | 1.374 | 1.393 | 1.363 | 3.607 |
| rate sec.beh (period 4) | 2.125 | 1.508 | NA | NA | NA | 2.589 | NA | 1.759 | NA |
| sec.beh linear shape | 0.291* | 0.371** | 0.087 | 0.256 | 0.092 | 0.604*** | 0.003 | 0.660** | 0.720* |
| sec.beh quadratic shape | 0.440* | 0.343† | 0.817† | 0.03 | 0.419 | 0.556** | 0.629† | 0.402* | 0.671† |
| sec.beh average similarity | 1.615* | 2.038* | 1.496 | 0.983 | 0.382 | 1.431 | 4.036† | 1.864† | 1.354 |
| sec.beh: effect from gender.covar | 0.04 | 0.006 | 0.324 | 0.171 | 0.366 | 0.072 | 0.283 | 0.272† | 0.277 |
| sec.beh: tot. sim. (secrecy) x ego's alc.beh | 0.005 | 0.105 | 0.28 | 0.39 | 0.175 | 0.162 | 0.602 | 0.432 | 0.41 |
| sec.beh: effect from meal.covar | 0.155 | 0.263 | 0.28 | 0.071 | 0.429 | 0.052 | 1.239 | 0.86 | 0.026 |
| rate con.beh (period 1) | 2.462 | 1.365 | 1.653 | 1.616 | 2.104 | 1.411 | 2.781 | 0.887 | 1.546 |
| rate con.beh (period 2) | 1.366 | 1.629 | NA | 1.023 | 1.245 | 1.741 | 1.363 | 0.661 | 1.289 |
| rate con.beh (period 3) | 1.203 | 1.213 | NA | 1.63 | NA | 0.882 | 1.058 | 0.922 | 1.447 |
| rate con.beh (period 4) | 1.232 | 1.629 | NA | NA | NA | 0.977 | NA | 1.175 | NA |
| con.beh linear shape | 0.237* | 0.446*** | 0.073 | 0.244 | 0.09 | 0.607** | 0.258 | 0.351† | 0.067 |
| con.beh quadratic shape | -0.240† | 0.2 | 0.201 | -0.597* | 0.249 | 0.046 | 0.237 | 0.341 | 0.351 |
| con.beh: effect from gender.covar | 0.096 | 0.281 | 0.385 | 0.789* | 0.073 | 0.410† | 0.495 | 0.912*** | 0.542 |
| con.beh: effect from alc.beh | -0.227* | -0.463** | 0.133 | -0.494† | 0.077 | -0.294* | 0.302 | -0.294† | -0.537† |
| con.beh: effect from meal.covar | 0.343 | 0.65 | 0.1 | 1.612 | 0.071 | 0.776 | 0.281 | 0.378 | 0.26 |
| Max Convergence | 0.25 | 0.24 | 0.22 | 0.21 | 0.24 | 0.2 | 0.23 | 0.22 | 0.23 |

N/A indicates parameters were excluded from the model

Table A4: Girls Schools: log odds of network dynamics (tie formation) or behaviour dynamics (step increase in behaviour level)

| Effect | Girls 1 | Girls 2 | Girls 3 | Girls 4 | Girls 5 | Girls 6 | Girls 7 | Girls 8 |
| --- | --- | --- | --- | --- | --- | --- | --- | --- |
| Network Dynamics |  |  |  |  |  |  |  |  |
| constant sch4nets rate (period 1) | 19.767 | 63.152 | 66.088 | 23.585 | 64.231 | 29.547 | 43.394 | 20.3 |
| constant sch4nets rate (period 2) | 18.615 | 36.015 | 21.791 | NA | 24.861 | 20.094 | 33.514 | 17.756 |
| constant sch4nets rate (period 3) | 21.158 | 47.53 | 26.849 | NA | 18.733 | 14.505 | 19.439 | 17.607 |
| constant sch4nets rate (period 4) | 10.517 | 20.704 | 21.532 | NA | 13.843 | NA | NA | 11.722 |
| outdegree (density) | -1.436*** | -1.897*** | -1.353*** | --1.915* | -1.464*** | -1.734*** | -2.767*** | -1.993*** |
| Reciprocity | 2.360*** | 2.884*** | 2.258*** | 2.568*** | 2.986*** | 2.295*** | 2.287*** | 2.213*** |
| transitive triplets | 0.432*** | 0.483*** | 0.417*** | 0.508*** | 0.533*** | 0.427*** | 0.406*** | 0.456*** |
| transitive recipr. Triplets | -0.255*** | -0.361*** | -0.241*** | 0.337 | -0.420*** | -0.281*** | -0.270*** | -0.290*** |
| indegree – popularity | 0.025** | 0.019 | 0.006 | 0.018 | 0.008 | 0.01 | 0.001 | 0 |
| outdegree – popularity | -0.146*** | 0.107 | -0.150*** | 0.069 | -0.095† | -0.096* | -0.083** | -0.100*** |
| outdegree – activity | 0.014 | 0.005 | 0.02 | 0.006 | 0.032 | 0.012 | 0.106*** | 0.030** |
| gender.dyadcovar | NA | NA | NA | NA | NA | NA | NA | NA |
| meal.dyadcovar | 0.116 | 0.108† | 0.041 | 0.285 | 0.023 | 0.148* | 0.124** | 0.116* |
| alc.beh alter | 0.085† | 0.109 | 0.065 | 0.084 | 0.002 | 0.172 | 0.057 | 0.025 |
| alc.beh ego | 0.029 | 0.148† | 0.101 | 0.094 | 0.275*** | 0.206 | 0.047 | 0.088 |
| alc.beh similarity | 0.245 | 0.496** | 0.191 | 0.109 | 0.375* | 0.13 | 0.109 | 0.22 |
| sec.beh alter | -0.129† | 0.088 | -0.172*** | 0.149 | 0.019 | 0.121 | 0.089 | 0.001 |
| sec.beh ego | -0.147† | 0.251** | 0.047 | 0.09 | 0.170† | 0.204 | 0.004 | 0.065 |
| sec.beh similarity | 0.459** | 0.143 | 0.367** | 0.356 | 0.22 | 0.268 | 0.16 | 0.206 |
| con.beh alter | 0.069 | -0.091† | 0.003 | 0.028 | 0.016 | 0.125 | 0.048 | 0.01 |
| con.beh ego | 0.084 | 0.001 | 0.105 | 0.452 | 0.136 | 0.041 | 0.16 | 0.052 |
| con.beh similarity | 0.200 | 0.042 | 0.138 | 0.372 | 0.255 | 0.152 | 0.544* | 0.067 |
| int. con.beh ego x alc.beh similarity | 0.111 | 0.351 | 0.101 | 2.159 | 0.185 | 1.21 | -0.763* | 0.608 |
| Behaviour Dynamics |  |  |  |  |  |  |  |  |
| rate alc.beh (period 1) | 1.39 | 2.803 | 1.853 | 1.135 | 1.369 | 5.205 | 2.357 | 2.685 |
| rate alc.beh (period 2) | 1.329 | 2.458 | 1.992 | NA | 1.346 | 1.646 | 2.148 | 1.637 |
| rate alc.beh (period 3) | 1.712 | 1.963 | 1.384 | NA | 1.693 | 2.118 | 2.216 | 1.767 |
| rate alc.beh (period 4) | 1.604 | 1.309 | 1.816 | NA | 1.07 | NA | NA | 1.455 |
| alc.beh linear shape | 0.697† | 0.391*** | 0.553** | 0.61 | 0.503** | 0.896** | 0.940*** | 0.770*** |
| alc.beh quadratic shape | 0.089 | 0.241** | 0.147 | 0.165 | 0.423** | 0.139 | 0.320* | 0.514** |
| alc.beh total similarity | 0.552* | 0.476*** | 0.281* | 0.303 | 0.574** | 0.364 | 0.329 | 0.929* |
| alc.beh: effect from gender.covar | NA | NA | NA | NA | NA | NA | NA | NA |
| alc.beh: effect from sec.beh | 0.146 | 0.496 | 0.366 | 1.111 | 0.685 | 0.672 | 0.072 | 0.14 |
| alc.beh: effect from con.beh | 1.211 | 0.254 | 0.653 | 0.408 | 0.169 | -1.272† | 0.564 | 0.72 |
| alc.beh: effect from meal.covar | 0.708 | 0.173 | 0.254 | 0.506 | 0.048 | 0.234 | 0.035 | 0.295 |
| alc.beh: effect from con.beh x alc.beh total similarity | 0.278 | 0.286 | 0.17 | 0.044 | 0.703* | 0.275 | 0.472 | 0.593 |
| alc.beh: effect from sec.beh x alc.beh total similarity | 0.259 | 0.051 | 0.272 | 0.14 | 0.006 | 0.929 | 0.138 | 0.574 |
| rate sec.beh (period 1) | 1.974 | 1.88 | 1.523 | 3.739 | 1.411 | 1.777 | 2.119 | 1.835 |
| rate sec.beh (period 2) | 1.967 | 2.168 | 2.054 | NA | 2.139 | 2.231 | 2.468 | 1.136 |
| rate sec.beh (period 3) | 1.923 | 1.695 | 1.848 | NA | 2.488 | 2.233 | 2.77 | 1.424 |
| rate sec.beh (period 4) | 0.981 | 1.861 | 1.445 | NA | 1.321 | NA | NA | 1.357 |
| sec.beh linear shape | 0.805*** | 0.434** | 0.431** | 1.447 | 0.879*** | 0.197 | 0.109 | 0.434** |
| sec.beh quadratic shape | 0.459* | 0.629** | 0.738*** | 0.816 | 0.559* | 0.012 | 0.188 | 0.561* |
| sec.beh average similarity | 0.076 | 1.856* | 2.604* | 1.302 | 0.281 | 0.639 | 0.557 | 1.243 |
| sec.beh: effect from gender.covar | NA | NA | NA | NA | NA | NA | NA | NA |
| sec.beh: tot. sim. (secrecy) x ego's alc.beh | -0.582† | 0.129 | 0.497† | NA | 0.517 | 0.912 | 0.301 | 0.443† |
| sec.beh: effect from meal.covar | 0.003 | 0.088 | 0.326 | 0.052 | 0.376 | 0.381 | 0.023 | 0.01 |
| rate con.beh (period 1) | 1.158 | 1.65 | 1.756 | 2.037 | 0.827 | 1.765 | 2.096 | 1.713 |
| rate con.beh (period 2) | 0.914 | 0.791 | 1.096 | NA | 1.713 | 1.519 | 2.107 | 2.221 |
| rate con.beh (period 3) | 0.783 | 1.091 | 1.103 | NA | 1.1 | 1.833 | 1.604 | 1.213 |
| rate con.beh (period 4) | 1.296 | 1.2 | 0.851 | NA | 1.316 | NA | NA | 0.831 |
| con.beh linear shape | 0.317 | 0.373** | 0.486* | 0.993 | 0.981*** | 0.091 | 0.128 | 0.333* |
| con.beh quadratic shape | -0.462† | 0.19 | 0.136 | 0.587 | 0.221 | -0.946* | 0.081 | 0.097 |
| con.beh: effect from gender.covar | NA | NA | NA | NA | NA | NA | NA | NA |
| con.beh: effect from alc.beh | 0.323 | -0.356* | 0.256 | 0.983 | -0.295† | -0.884† | 0.115 | 0.071 |
| con.beh: effect from meal.covar | 0.25 | 0.291 | 0.042 | 1.182 | -1.212† | 0.047 | 0.239 | -0.449† |
| Max Convergence | 0.24 | 0.24 | 0.23 | 0.19 | 0.24 | 0.19 | 0.23 | 0.21 |

N/A indicates parameters were excluded from the model

Table A5: Boys Schools: log odds of network dynamics (tie formation) or behaviour dynamics (step increase in behaviour level)

| Effect | Boys 1 | Boys 2 | Boys 3 | Boys 4 | Boys 5 |
| --- | --- | --- | --- | --- | --- |
| Network Dynamics |  |  |  |  |  |
| constant sch4nets rate (period 1) | 19.679 | 25.13 | 30.899 | 23.292 | 16.412 |
| constant sch4nets rate (period 2) | NA | 22.377 | 19.959 | 23.488 | 14.723 |
| constant sch4nets rate (period 3) | NA | 22.242 | 116.666 | 44.548 | 19.273 |
| constant sch4nets rate (period 4) | NA | NA | 14.14 | NA | 11.828 |
| outdegree (density) | -0.732 | -2.034*** | -2.083*** | -1.833*** | -1.569*** |
| Reciprocity | 1.967*** | 2.556*** | 2.223*** | 2.339*** | 2.247*** |
| transitive triplets | 0.587*** | 0.614*** | 0.438*** | 0.519*** | 0.448*** |
| transitive recipr. Triplets | -0.348** | -0.415*** | -0.302*** | -0.411*** | -0.275*** |
| indegree – popularity | 0.008 | 0.029*** | 0.013* | 0.009 | 0.029*** |
| outdegree – popularity | 0.197 | -0.155*** | -0.122*** | -0.142*** | -0.150*** |
| outdegree – activity | 0.052 | 0.003 | 0.051*** | 0.026** | 0.009 |
| gender.dyadcovar | NA | NA | NA | NA | NA |
| meal.dyadcovar | 0.027 | 0.03 | 0.108* | 0.043 | 0.316*** |
| alc.beh alter | 0.134 | 0.000 | 0.018 | 0.086 | 0.014 |
| alc.beh ego | 0.064 | 0.012 | 0.003 | 0.056 | -0.194** |
| alc.beh similarity | 0.443 | 0.244 | 0.406*** | 0.394* | 0.232 |
| sec.beh alter | 0.049 | 0.080 | 0.021 | 0.053 | -0.157** |
| sec.beh ego | 0.540 | 0.015 | 0.014 | 0.043 | -0.161† |
| sec.beh similarity | 0.835 | 0.314† | 0.230† | 0.122 | 0.066 |
| con.beh alter | 0.151 | 0.057 | 0.014 | 0.031 | 0.095 |
| con.beh ego | 0.499 | 0.075 | 0.066 | 0.003 | 0.022 |
| con.beh similarity | 0.496 | 0.26 | 0.061 | 0.083 | 0.243 |
| int. con.beh ego x alc.beh similarity | 1.334 | 0.464 | 0.075 | 0.455 | 0.470 |
| Behaviour Dynamics |  |  |  |  |  |
| rate alc.beh (period 1) | 2.701 | 1.992 | 1.700 | 2.075 | 2.782 |
| rate alc.beh (period 2) | NA | 2.803 | 1.707 | 2.089 | 1.999 |
| rate alc.beh (period 3) | NA | 2.367 | 2.118 | 2.422 | 2.391 |
| rate alc.beh (period 4) | NA | NA | 1.632 | NA | 2.39 |
| alc.beh linear shape | 0.074 | 0.180* | 0.277** | 0.395† | 0.218* |
| alc.beh quadratic shape | 0.219 | 0.288** | 0.253** | 0.387** | 0.264* |
| alc.beh total similarity | 0.551 | 0.400** | 0.283† | 0.495† | 0.557*** |
| alc.beh: effect from gender.covar | NA | NA | NA | NA | NA |
| alc.beh: effect from sec.beh | 0.597 | 0.033 | -0.484* | 0.007 | 0.183 |
| alc.beh: effect from con.beh | 0.509 | 0.189 | 0.233 | 0.704 | 0.458 |
| alc.beh: effect from meal.covar | 0.15 | 0.111 | 0.722† | 0.026 | 0.364 |
| alc.beh: effect from con.beh x alc.beh total similarity | 0.246 | 0.009 | 0.458 | 0.524 | 0.181 |
| alc.beh: effect from sec.beh x alc.beh total similarity | 0.568 | 0.179 | 0.311 | 0.588 | 0.093 |
| rate sec.beh (period 1) | 3.685 | 2.299 | 2.348 | 3.422 | 1.621 |
| rate sec.beh (period 2) | NA | 2.162 | 2.665 | 3.298 | 1.638 |
| rate sec.beh (period 3) | NA | 2.43 | 1.81 | 2.695 | 1.399 |
| rate sec.beh (period 4) | NA | NA | 1.826 | NA | 1.445 |
| sec.beh linear shape | 1.119 | 0.293* | 0.177* | 0.569** | 0.208† |
| sec.beh quadratic shape | 0.096 | 0.191 | 0.309 | 0.21 | 0.036 |
| sec.beh average similarity | 1.513 | 1.632 | 0.797 | 1.341 | 0.965 |
| sec.beh: effect from gender.covar | NA | NA | NA | NA | NA |
| sec.beh: tot. sim. (secrecy) x ego's alc.beh | 2.473 | 0.489 | 0.194 | 1.006 | 0.363 |
| sec.beh: effect from meal.covar | 0.741 | 0.044 | 0.21 | 0.106 | 0.04 |
| rate con.beh (period 1) | 2.88 | 1.806 | 2.271 | 1.748 | 1.273 |
| rate con.beh (period 2) | NA | 1.741 | 1.655 | 1.314 | 1.554 |
| rate con.beh (period 3) | NA | 1.098 | 1.891 | 1.688 | 1.352 |
| rate con.beh (period 4) | NA | NA | 1.003 | NA | 2.18 |
| con.beh linear shape | 0.317 | 0.105 | 0.292* | 0.01 | 0.051 |
| con.beh quadratic shape | 0.171 | -0.303† | 0.012 | 0.322 | -0.477** |
| con.beh: effect from gender.covar | NA | NA | NA | NA | NA |
| con.beh: effect from alc.beh | 0.115 | -0.308† | 0.13 | -0.413* | 0.199 |
| con.beh: effect from meal.covar | 0.021 | 0.051 | 0.171 | 0.166 | 0.369 |
| Max Convergence | 0.19 | 0.22 | 0.21 | 0.23 | 0.24 |

N/A indicates parameters were excluded from the model

Table A6: Random effects meta-analysis of network parameters across schools

| Effect | Estimates | Lower Cl | Upper Cl | Standard error |
| --- | --- | --- | --- | --- |
| constant net.list rate (period 1) | 28.84 | 21.89 | 35.8 | 3.55 |
| constant net.list rate (period 2) | 21.47 | 18.84 | 24.11 | 1.35 |
| constant net.list rate (period 3) | 28.72 | 20.51 | 36.93 | 4.19 |
| constant net.list rate (period 4) | 16.35 | 13.79 | 18.91 | 1.31 |
| outdegree (density) | -1.97 | -2.13 | -1.81 | 0.08 |
| Reciprocity | 2.36 | 2.26 | 2.45 | 0.05 |
| transitive triplets | 0.47 | 0.44 | 0.49 | 0.01 |
| transitive reciprocated triplets | -0.32 | -0.35 | -0.3 | 0.01 |
| indegree – popularity | 0.02 | 0.01 | 0.02 | 0.00 |
| outdegree – popularity | -0.12 | -0.13 | -0.11 | 0.01 |
| outdegree – activity | 0.02 | 0.01 | 0.04 | 0.01 |
| gender.dyad | 0.29 | 0.21 | 0.38 | 0.04 |
| meal.dyad | 0.08 | 0.02 | 0.13 | 0.03 |
| alc.beh alter | 0.03 | 0.01 | 0.05 | 0.01 |
| alc.beh ego | 0.01 | -0.03 | 0.05 | 0.02 |
| alc.beh similarity | 0.35 | 0.27 | 0.43 | 0.04 |
| sec.beh alter | -0.05 | -0.08 | -0.02 | 0.01 |
| sec.beh ego | 0.00 | -0.05 | 0.05 | 0.03 |
| sec.beh similarity | 0.09 | 0.00 | 0.18 | 0.05 |
| con.beh alter | -0.01 | -0.03 | 0.02 | 0.01 |
| con.beh ego | -0.02 | -0.07 | 0.02 | 0.02 |
| con.beh similarity | 0.12 | 0.03 | 0.20 | 0.04 |
| con.beh ego x alc.beh similarity | 0.09 | -0.12 | 0.29 | 0.1 |
| rate alc.beh (period 1) | 1.67 | 1.46 | 1.88 | 0.11 |
| rate alc.beh (period 2) | 1.60 | 1.41 | 1.79 | 0.10 |
| rate alc.beh (period 3) | 1.53 | 1.31 | 1.74 | 0.11 |
| rate alc.beh (period 4) | 1.24 | 1.07 | 1.4 | 0.08 |
| alc.beh linear shape | 0.46 | 0.35 | 0.56 | 0.05 |
| alc.beh quadratic shape | 0.26 | 0.2 | 0.31 | 0.03 |
| alc.beh total similarity | 0.46 | 0.38 | 0.54 | 0.04 |
| alc.beh: effect from gender.covar | 0.14 | 0.00 | 0.29 | 0.08 |
| alc.beh: effect from sec.beh | -0.22 | -0.35 | -0.09 | 0.07 |
| alc.beh: effect from con.beh | -0.31 | -0.46 | -0.17 | 0.07 |
| alc.beh: effect from fmeals.covar | 0.06 | -0.09 | 0.22 | 0.08 |
| alc.beh: effect from con.beh x alc.beh total similarity | 0.1 | -0.09 | 0.3 | 0.1 |
| alc.beh: effect from sec.beh x alc.beh total similarity | 0.08 | -0.07 | 0.24 | 0.08 |
| rate sec.beh (period 1) | 1.81 | 1.59 | 2.03 | 0.11 |
| rate sec.beh (period 2) | 1.74 | 1.52 | 1.96 | 0.11 |
| rate sec.beh (period 3) | 1.59 | 1.4 | 1.78 | 0.1 |
| rate sec.beh (period 4) | 1.54 | 1.32 | 1.76 | 0.11 |
| sec.beh linear shape | 0.34 | 0.22 | 0.46 | 0.06 |
| sec.beh quadratic shape | 0.45 | 0.34 | 0.57 | 0.06 |
| sec.beh average similarity | 1.2 | 0.65 | 1.75 | 0.28 |
| sec.beh: effect from gender.covar | 0.02 | -0.11 | 0.16 | 0.07 |
| sec.beh: alter's (net.list) alc.beh average | -0.04 | -0.2 | 0.11 | 0.08 |
| sec.beh: effect from fmeals.covar | -0.1 | -0.24 | 0.03 | 0.07 |
| rate con.beh (period 1) | 1.43 | 1.24 | 1.63 | 0.1 |
| rate con.beh (period 2) | 1.23 | 1.04 | 1.41 | 0.09 |
| rate con.beh (period 3) | 1.12 | 0.99 | 1.26 | 0.07 |
| rate con.beh (period 4) | 1.09 | 0.93 | 1.26 | 0.08 |
| con.beh linear shape | 0.25 | 0.16 | 0.35 | 0.05 |
| con.beh quadratic shape | -0.23 | -0.33 | -0.14 | 0.05 |
| con.beh: effect from gender.covar | 0.38 | 0.19 | 0.58 | 0.1 |
| con.beh: effect from alc.beh | -0.25 | -0.33 | -0.18 | 0.04 |
| con.beh: effect from fmeals.covar | -0.16 | -0.32 | 0.00 | 0.08 |

Meta analysis parameter: the pooled parameter value across all schools in the sample

Table A7: Metaregression of network parameters including school gender and proportion of frequent drinkers in year three

| Effect | Intercept | Boys | Girls | % Frequent Drinkers |
| --- | --- | --- | --- | --- |
| constant net.list rate (period 1) | **24.86 (5.84, 43.87)   p=0.01** | -0.45 (-17.45, 16.55)   p=0.96 | **12.91 (-1.85, 27.66)   p=0.09** | -2.11 (-84.11, 79.89)   p=0.96 |
| constant net.list rate (period 2) | **18.89 (10.44, 27.33)   p=<0.001** | -1.74 (-8.86, 5.38)   p=0.63 | 1.17 (-5.55, 7.89)   p=0.73 | 11.61 (-28.16, 51.37)   p=0.57 |
| constant net.list rate (period 3) | **36.85 (18.42, 55.29)   p=<0.001** | 9.22 (-8.49, 26.93)   p=0.31 | -2.01 (-17.09, 13.07)   p=0.79 | -45.28 (-134.36, 43.81)   p=0.32 |
| constant net.list rate (period 4) | **17.47 (8.17, 26.77)   p=<0.001** | **-7.28 (-14.97, 0.42)   p=0.06** | -4.44 (-9.91, 1.02)   p=0.11 | 11.19 (-37.86, 60.24)   p=0.65 |
| outdegree (density) | **-1.98 (-2.49, -1.46)   p=<0.001** | 0.28 (-0.17, 0.72)   p=0.22 | **0.33 (-0.06, 0.73)   p=0.1** | -0.76 (-3.09, 1.56)   p=0.52 |
| Reciprocity | **2.46 (2.15, 2.78)   p=<0.001** | 0.12 (-0.15, 0.38)   p=0.38 | **0.23 (0.00, 0.47)   p=0.05** | -0.97 (-2.48, 0.55)   p=0.21 |
| transitive triplets | **0.47 (0.39, 0.54)   p=<0.001** | 0.04 (-0.03, 0.11)   p=0.24 | -0.01 (-0.07, 0.05)   p=0.72 | -0.01 (-0.37, 0.35)   p=0.94 |
| transitive reciprocated triplets | **-0.34 (-0.43, -0.25)   p=<0.001** | -0.02 (-0.10, 0.06)   p=0.62 | 0.03 (-0.04, 0.10)   p=0.42 | 0.04 (-0.39, 0.47)   p=0.85 |
| indegree - popularity | **0.03 (0.01, 0.05)   p=<0.001** | **0.01 (0.00, 0.03)   p=0.09** | 0.00 (-0.01, 0.01)   p=0.98 | **-0.09 (-0.19, 0.00)   p=0.05** |
| outdegree - popularity | **-0.12 (-0.15, -0.08)   p=<0.001** | **-0.04 (-0.07, -0.01)   p=0.01** | -0.02 (-0.05, 0.01)   p=0.12 | 0.06 (-0.13, 0.26)   p=0.52 |
| outdegree - activity | 0.01 (-0.03, 0.05)   p=0.53 | -0.02 (-0.05, 0.02)   p=0.35 | -0.02 (-0.05, 0.01)   p=0.13 | 0.1 (-0.07, 0.28)   p=0.25 |
| gender.dyad | 0.01 (-0.28, 0.29)   p=0.97 |  |  | **1.53 (0.08, 2.98)   p=0.04** |
| meal.dyad | 0.01 (-0.16, 0.17)   p=0.95 | 0.04 (-0.09, 0.18)   p=0.53 | 0.03 (-0.1, 0.15)   p=0.69 | 0.21 (-0.51, 0.94)   p=0.56 |
| alc.beh alter | 0.05 (-0.03, 0.13)   p=0.25 | -0.05 (-0.11, 0.01)   p=0.12 | 0 (-0.06, 0.05)   p=0.93 | -0.02 (-0.42, 0.38)   p=0.93 |
| alc.beh ego | 0.1 (-0.03, 0.24)   p=0.14 | -0.01 (-0.12, 0.09)   p=0.79 | 0.06 (-0.04, 0.16)   p=0.23 | -0.5 (-1.17, 0.17)   p=0.14 |
| alc.beh similarity | **0.60 (0.31, 0.89)   p=<0.001** | 0.04 (-0.19, 0.27)   p=0.73 | -0.08 (-0.28, 0.11)   p=0.40 | -1.11 (-2.55, 0.33)   p=0.13 |
| sec.beh alter | -0.10 (-0.20, 0.01)   p=0.07 | **-0.08 (-0.16, 0.00)   p=0.05** | **-0.07 (-0.14, 0.01)   p=0.07** | 0.38 (-0.12, 0.88)   p=0.14 |
| sec.beh ego | 0.03 (-0.17, 0.24)   p=0.75 | -0.03 (-0.2, 0.14)   p=0.72 | 0.02 (-0.12, 0.16)   p=0.81 | -0.17 (-1.14, 0.81)   p=0.74 |
| sec.beh similarity | 0.13 (-0.24, 0.5)   p=0.50 | 0.09 (-0.22, 0.39)   p=0.58 | 0.00 (-0.25, 0.25)   p=1.00 | -0.28 (-2.02, 1.46)   p=0.75 |
| con.beh alter | -0.06 (-0.17, 0.04)   p=0.24 | 0.06 (-0.02, 0.14)   p=0.14 | 0.00 (-0.07, 0.07)   p=0.93 | 0.21 (-0.32, 0.74)   p=0.44 |
| con.beh ego | **0.15 (-0.03, 0.33)   p=0.1** | 0.00 (-0.14, 0.14)   p=0.99 | -0.01 (-0.14, 0.13)   p=0.93 | **-0.80 (-1.67, 0.06)   p=0.07** |
| con.beh similarity | 0.12 (-0.18, 0.42)   p=0.43 | -0.17 (-0.42, 0.07)   p=0.17 | -0.01 (-0.23, 0.21)   p=0.92 | 0.20 (-1.29, 1.70)   p=0.79 |
| con.beh ego x alc.beh similarity | 0.20 (-0.50, 0.90)   p=0.57 | -0.3 (-0.87, 0.27)   p=0.30 | -0.21 (-0.72, 0.30)   p=0.42 | 0.04 (-3.50, 3.58)   p=0.98 |
| rate alc.beh (period 1) | **0.76 (0.12, 1.41)   p=0.02** | 0.15 (-0.48, 0.78)   p=0.64 | 0.19 (-0.27, 0.64)   p=0.43 | **4.14 (0.50, 7.78)   p=0.03** |
| rate alc.beh (period 2) | **0.98 (0.49, 1.46)   p=<0.001** | **0.48 (-0.03, 0.99)   p=0.07** | 0.14 (-0.22, 0.51)   p=0.44 | **2.29 (-0.22, 4.81)   p=0.07** |
| rate alc.beh (period 3) | **1.23 (0.68, 1.77)   p=<0.001** | **1.17 (0.51, 1.83)   p=<0.001** | **0.57 (0.18, 0.96)   p=<0.001** | -0.28 (-3.07, 2.52)   p=0.85 |
| rate alc.beh (period 4) | **1.17 (0.51, 1.83)   p=<0.001** | **0.70 (-0.07, 1.47)   p=0.07** | 0.23 (-0.12, 0.57)   p=0.20 | -0.33 (-3.89, 3.22)   p=0.85 |
| alc.beh linear shape | 0.16 (-0.12, 0.45)   p=0.26 | -0.50 (-0.70, -0.29)   p=<0.001 | -0.1 (-0.31, 0.12)   p=0.37 | 2.25 (0.80, 3.7)   p=<0.001 |
| alc.beh quadratic shape | **0.21 (0.01, 0.41)   p=0.04** | 0.02 (-0.13, 0.18)   p=0.77 | -0.01 (-0.14, 0.13)   p=0.94 | 0.21 (-0.77, 1.19)   p=0.68 |
| alc.beh total similarity | **0.63 (0.34, 0.92)   p=<0.001** | -0.01 (-0.25, 0.24)   p=0.96 | -0.01 (-0.21, 0.19)   p=0.9 | -0.83 (-2.38, 0.72)   p=0.30 |
| alc.beh: effect from gender.covar | -0.04 (-0.62, 0.53)   p=0.88 |  |  | 1.06 (-2.05, 4.18)   p=0.50 |
| alc.beh: effect from sec.beh | -0.31 (-0.88, 0.26)   p=0.28 | 0.1 (-0.22, 0.42)   p=0.54 | 0.11 (-0.23, 0.44)   p=0.54 | 0.13 (-2.4, 2.66)   p=0.92 |
| alc.beh: effect from con.beh | -0.12 (-0.77, 0.53)   p=0.71 | 0.18 (-0.23, 0.58)   p=0.39 | 0.06 (-0.36, 0.47)   p=0.78 | -1.28 (-4.36, 1.79)   p=0.41 |
| alc.beh: effect from fmeals.covar | -0.51 (-1.17, 0.14)   p=0.12 | 0.03 (-0.36, 0.42)   p=0.88 | -0.12 (-0.57, 0.34)   p=0.62 | **2.43 (-0.29, 5.14)   p=0.08** |
| alc.beh: effect from con.beh x alc.beh total similarity | -0.28 (-0.98, 0.43)   p=0.44 | 0.2 (-0.37, 0.77)   p=0.48 | **0.41 (-0.08, 0.91)   p=0.10** | 0.94 (-2.86, 4.73)   p=0.63 |
| alc.beh: effect from sec.beh x alc.beh total similarity | -0.34 (-0.88, 0.19)   p=0.21 | 0.11 (-0.34, 0.56)   p=0.63 | 0.06 (-0.31, 0.43)   p=0.75 | 1.81 (-0.87, 4.48)   p=0.19 |
| rate sec.beh (period 1) | **1.69 (0.97, 2.42)   p=<0.001** | 0.12 (-0.55, 0.8)   p=0.73 | -0.18 (-0.67, 0.31)   p=0.48 | 0.75 (-2.56, 4.05)   p=0.66 |
| rate sec.beh (period 2) | **1.91 (1.02, 2.79)   p=<0.001** | 0.43 (-0.28, 1.14)   p=0.23 | 0.02 (-0.55, 0.59)   p=0.95 | -0.93 (-5.02, 3.17)   p=0.66 |
| rate sec.beh (period 3) | **1.36 (0.64, 2.07)   p=<0.001** | 0.33 (-0.23, 0.88)   p=0.25 | 0.33 (-0.18, 0.85)   p=0.2 | 0.46 (-3.23, 4.15)   p=0.81 |
| rate sec.beh (period 4) | **1.44 (0.6, 2.28)   p=<0.001** | -0.42 (-1.17, 0.33)   p=0.27 | -0.55 (-1.08, -0.03)   p=0.04 | 2.4 (-2.14, 6.94)   p=0.3 |
| sec.beh linear shape | **0.9 (0.62, 1.18)   p=<0.001** | 0.1 (-0.08, 0.29)   p=0.28 | **0.22 (0.03, 0.41)   p=0.02** | **-2.94 (-4.16, -1.73)   p=<0.001** |
| sec.beh quadratic shape | **0.42 (0.02, 0.83)   p=0.04** | -0.36 (-0.76, 0.04)   p=0.08 | 0.11 (-0.15, 0.37)   p=0.42 | 0.17 (-1.84, 2.18)   p=0.87 |
| sec.beh average similarity | **1.7 (-0.22, 3.62)   p=0.08** | -2.06 (-3.92, -0.2)   p=0.03 | -0.41 (-1.63, 0.82)   p=0.52 | -0.4 (-9.8, 9.01)   p=0.93 |
| sec.beh: effect from gender.covar | 0.41 (-0.14, 0.96)   p=0.14 |  |  | -2.22 (-5.25, 0.82)   p=0.15 |
| sec.beh: alter's (net.list) alc.beh average | **-0.67 (-1.17, -0.16)   p=0.01** | -0.44 (-0.98, 0.1)   p=0.11 | 0.15 (-0.18, 0.49)   p=0.36 | 2.81 (0.38, 5.23)   p=0.02 |
| sec.beh: effect from fmeals.covar | -0.07 (-0.59, 0.46)   p=0.80 | 0.06 (-0.3, 0.42)   p=0.76 | 0.07 (-0.33, 0.46)   p=0.74 | -0.33 (-2.53, 1.87)   p=0.77 |
| rate con.beh (period 1) | **0.58 (0.09, 1.07)   p=0.02** | -0.02 (-0.53, 0.5)   p=0.95 | -0.24 (-0.66, 0.18)   p=0.27 | 4.56 (1.94, 7.18)   p=0 |
| rate con.beh (period 2) | 0.3 (-0.11, 0.71)   p=0.16 | 0.19 (-0.27, 0.66)   p=0.41 | -0.06 (-0.39, 0.27)   p=0.73 | 4.28 (1.99, 6.57)   p=<0.001 |
| rate con.beh (period 3) | **0.57 (0.08, 1.07)   p=0.02** | 0.07 (-0.33, 0.47)   p=0.73 | -0.1 (-0.41, 0.22)   p=0.55 | 2.83 (0.31, 5.35)   p=0.03 |
| rate con.beh (period 4) | **1.58 (0.94, 2.22)   p=<0.001** | 0.18 (-0.45, 0.80)   p=0.58 | -0.01 (-0.4, 0.37)   p=0.94 | -2.45 (-5.9, 0.99)   p=0.16 |
| con.beh linear shape | **0.58 (0.31, 0.84)   p=<0.001** | -0.09 (-0.27, 0.09)   p=0.33 | **0.17 (-0.02, 0.35)   p=0.08** | **-1.53 (-2.68, -0.38)   p=0.01** |
| con.beh quadratic shape | -0.29 (-0.64, 0.06)   p=0.11 | -0.04 (-0.29, 0.21)   p=0.76 | 0.09 (-0.15, 0.33)   p=0.45 | 0.16 (-1.44, 1.77)   p=0.84 |
| con.beh: effect from gender.covar | **1.08 (0.46, 1.7)   p<0.001** |  |  | **-3.67 (-6.73, -0.62)   p=0.02** |
| con.beh: effect from alc.beh | **-0.54 (-0.81, -0.27)   p=<0.001** | -0.01 (-0.21, 0.18)   p=0.89 | 0 (-0.18, 0.17)   p=0.97 | **1.28 (0.01, 2.54)   p=0.05** |
| con.beh: effect from fmeals.covar | -0.27 (-0.97, 0.43)   p=0.45 | -0.07 (-0.48, 0.35)   p=0.75 | -0.36 (-0.82, 0.1)   p=0.13 | 1.02 (-1.79, 3.84)   p=0.48 |

**Bold type** denotes p value below 0.1

Meta-regression intercept: the estimated parameter value for a coeducational school with the mean proportion of frequent drinkers in the study sample

Table A8: Metaregression of school gender and proportion of frequent drinkers on research question parameters

| Effect | Intercept | Boys | Girls | % Frequent Drinkers |
| --- | --- | --- | --- | --- |
| alc.beh alter | 0.05 (-0.03, 0.13)   p=0.25 | -0.05 (-0.11, 0.01)   p=0.12 | 0 (-0.06, 0.05)   p=0.93 | -0.02 (-0.42, 0.38)   p=0.93 |
| alc.beh ego | 0.1 (-0.03, 0.24)   p=0.14 | -0.01 (-0.12, 0.09)   p=0.79 | 0.06 (-0.04, 0.16)   p=0.23 | -0.5 (-1.17, 0.17)   p=0.14 |
| alc.beh similarity | **0.60 (0.31, 0.89)   p=<0.001** | 0.04 (-0.19, 0.27)   p=0.73 | -0.08 (-0.28, 0.11)   p=0.40 | -1.11 (-2.55, 0.33)   p=0.13 |
| sec.beh alter | -0.10 (-0.20, 0.01)   p=0.07 | **-0.08 (-0.16, 0.00)   p=0.05** | **-0.07 (-0.14, 0.01)   p=0.07** | 0.38 (-0.12, 0.88)   p=0.14 |
| sec.beh ego | 0.03 (-0.17, 0.24)   p=0.75 | -0.03 (-0.2, 0.14)   p=0.72 | 0.02 (-0.12, 0.16)   p=0.81 | -0.17 (-1.14, 0.81)   p=0.74 |
| sec.beh similarity | 0.13 (-0.24, 0.5)   p=0.50 | 0.09 (-0.22, 0.39)   p=0.58 | 0.00 (-0.25, 0.25)   p=1.00 | -0.28 (-2.02, 1.46)   p=0.75 |
| con.beh alter | -0.06 (-0.17, 0.04)   p=0.24 | 0.06 (-0.02, 0.14)   p=0.14 | 0.00 (-0.07, 0.07)   p=0.93 | 0.21 (-0.32, 0.74)   p=0.44 |
| con.beh ego | **0.15 (-0.03, 0.33)   p=0.1** | 0.00 (-0.14, 0.14)   p=0.99 | -0.01 (-0.14, 0.13)   p=0.93 | **-0.80 (-1.67, 0.06)   p=0.07** |
| con.beh similarity | 0.12 (-0.18, 0.42)   p=0.43 | -0.17 (-0.42, 0.07)   p=0.17 | -0.01 (-0.23, 0.21)   p=0.92 | 0.20 (-1.29, 1.70)   p=0.79 |
| con.beh ego x alc.beh similarity | 0.20 (-0.50, 0.90)   p=0.57 | -0.3 (-0.87, 0.27)   p=0.30 | -0.21 (-0.72, 0.30)   p=0.42 | 0.04 (-3.50, 3.58)   p=0.98 |
| alc.beh total similarity | **0.63 (0.34, 0.92)   p=<0.001** | -0.01 (-0.25, 0.24)   p=0.96 | -0.01 (-0.21, 0.19)   p=0.9 | -0.83 (-2.38, 0.72)   p=0.30 |
| alc.beh: effect from sec.beh | -0.31 (-0.88, 0.26)   p=0.28 | 0.1 (-0.22, 0.42)   p=0.54 | 0.11 (-0.23, 0.44)   p=0.54 | 0.13 (-2.4, 2.66)   p=0.92 |
| alc.beh: effect from con.beh | -0.12 (-0.77, 0.53)   p=0.71 | 0.18 (-0.23, 0.58)   p=0.39 | 0.06 (-0.36, 0.47)   p=0.78 | -1.28 (-4.36, 1.79)   p=0.41 |
| alc.beh: effect from con.beh x alc.beh total similarity | -0.28 (-0.98, 0.43)   p=0.44 | 0.20 (-0.37, 0.77)   p=0.48 | **0.41 (-0.08, 0.91)   p=0.10** | 0.94 (-2.86, 4.73)   p=0.63 |
| alc.beh: effect from sec.beh x alc.beh total similarity | -0.34 (-0.88, 0.19)   p=0.21 | 0.11 (-0.34, 0.56)   p=0.63 | 0.06 (-0.31, 0.43)   p=0.75 | 1.81 (-0.87, 4.48)   p=0.19 |
| sec.beh average similarity | **1.7 (-0.22, 3.62)   p=0.08** | -2.06 (-3.92, -0.2)   p=0.03 | -0.41 (-1.63, 0.82)   p=0.52 | -0.4 (-9.8, 9.01)   p=0.93 |
| sec.beh: alter's (net.list) alc.beh average | **-0.67 (-1.17, -0.16)   p=0.01** | -0.44 (-0.98, 0.1)   p=0.11 | 0.15 (-0.18, 0.49)   p=0.36 | 2.81 (0.38, 5.23)   p=0.02 |
| con.beh: effect from alc.beh | **-0.54 (-0.81, -0.27)   p=<0.001** | -0.01 (-0.21, 0.18)   p=0.89 | 0.00 (-0.18, 0.17)   p=0.97 | **1.28 (0.01, 2.54)   p=0.05** |

**Bold type** denotes p value below 0.1

Meta-regression intercept: the estimated parameter value for a coeducational school with the mean proportion of frequent drinkers in the study sample

Time trends were assessed using score type tests within each school. The p values from score type tests were combined using: mean; logit; sum of logs; vote counting, and Schweder graph methods. We concluded that there was evidence for a time trend where three or more tests identified a trend. Tests were conducted using the R package metap.

Table A9: Results of five p value meta-analyses for time trend score type tests

|  | Logit | Mean | Sum of logs | Vote counting | Schweder graph | Evidence of time trend |
| --- | --- | --- | --- | --- | --- | --- |
| siena.alc.beh[[i]] alter | **<0.001** | **<0.001** | **<0.001** | **0.004** | **Large Curve** | **Yes** |
| siena.alc.beh[[i]] ego | **0.001** | **0.014** | **0.001** | **0.119** | **Slight Curve** | **Yes** |
| siena.alc.beh[[i]] similarity | 0.085 | 0.051 | 0.136 | 0.004 | Slight Curve | No |
| siena.sec.beh[[i]] alter | 0.008 | 0.237 | <0.001 | 0.407 | Minimal Curve | No |
| siena.sec.beh[[i]] ego | **0.001** | **0.041** | **<0.001** | **0.119** | **No Curve** | **Yes** |
| siena.sec.beh[[i]] similarity | **0.008** | **0.016** | **0.011** | **0.24** | **No Curve** | **Yes** |
| siena.con.beh[[i]] alter | 0.462 | 0.194 | 0.243 | 0.407 | No Curve | No |
| siena.con.beh[[i]] ego | **0.002** | **0.002** | **0.003** | **0.015** | **Large Curve** | **Yes** |
| siena.con.beh[[i]] similarity | 0.104 | 0.189 | 0.085 | 0.76 | No Curve | No |
| siena.con.beh[[i]] ego x siena.alc.beh[[i]] similarity | **0.014** | **0.023** | **0.004** | **0.048** | **Large Curve** | **Yes** |
| behavior siena.alc.beh[[i]] total similarity | 0.081 | 0.021 | 0.162 | 0.015 | Slight Curve | No |
| behavior siena.alc.beh[[i]]: effect from siena.sec.beh[[i]] | 0.606 | 0.605 | 0.759 | 0.593 | No Curve | No |
| behavior siena.alc.beh[[i]]: effect from siena.con.beh[[i]] | 0.968 | 0.857 | 0.937 | 0.881 | No Curve | No |
| behavior siena.alc.beh[[i]]: effect from siena.con.beh[[i]] x behavior siena.alc.beh[[i]] total similarity | **0.011** | **0.01** | **0.031** | **0.015** | **Slight Curve** | **Yes** |
| behavior siena.alc.beh[[i]]: effect from siena.sec.beh[[i]] x behavior siena.alc.beh[[i]] total similarity | 0.883 | 0.705 | 0.345 | 0.593 | No Curve | No |
| behavior siena.sec.beh[[i]] average similarity | 0.117 | 0.133 | 0.1 | 0.593 | No Curve | No |
| behavior siena.sec.beh[[i]]: alter's (siena.net.list[[i]]) siena.alc.beh[[i]] average | 0.231 | 0.292 | 0.252 | 0.24 | No Curve | No |
| behavior siena.con.beh[[i]]: effect from siena.alc.beh[[i]] | 0.244 | 0.359 | 0.208 | 0.593 | No Curve | No |

Results based on 18 schools with three or more waves of data
